# CRAFT (Cerclage after full dilatation caesarean section): protocol of a mixed methods study investigating the role of previous in-labour caesarean section in preterm birth risk

**DOI:** 10.1186/s12884-020-03375-z

**Published:** 2020-11-16

**Authors:** Naomi Carlisle, Agnieszka Glazewska-Hallin, Lisa Story, Jenny Carter, Paul T. Seed, Natalie Suff, Lucie Giblin, Jana Hutter, Raffaele Napolitano, Mary Rutherford, Daniel C. Alexander, Nigel Simpson, Amrita Banerjee, Anna L. David, Andrew H. Shennan

**Affiliations:** 1 Department of Women and Children’s Health, School of Life Course Sciences, King’s College London, 10th Floor, North Wing, St Thomas’ Hospital, Westminster Bridge Road, London, SE1 7EH UK; 2 Centre for the Developing Brain, King’s College London, 1st Floor South Wing, St Thomas’ Hospital, London, SE1 7EH UK; 3 Elizabeth Garrett Anderson Institute for Women’s Health, University College London, Room 244, Medical School Building, Huntley Street, London, WC1E 6AU UK; 4 Department of Computer Science, University College London, Gower Street, London, WC1E 6BT UK; 5 Delivery Suite, C Floor, Clarendon Wing, The General Infirmary at Leeds, Belmont Grove, Leeds, LS2 9NS UK; 6 NIHR University College London Hospitals Biomedical Research Centre, 149 Tottenham Court Road, London, W1T 7DN UK

**Keywords:** Caesarean section, Fully dilated, Full dilatation, Labour caesarean, Preterm birth, Late miscarriage, Cervix, Delivery

## Abstract

**Background:**

Full dilatation caesarean sections are associated with recurrent early spontaneous preterm birth and late miscarriage. The risk following first stage caesarean sections, are less well defined, but appears to be increased in late-first stage of labour. The mechanism for this increased risk of late miscarriage and early spontaneous preterm birth in these women is unknown and there are uncertainties with regards to clinical management. Current predictive models of preterm birth (based on transvaginal ultrasound and quantitative fetal fibronectin) have not been validated in these women and it is unknown whether the threshold to define a short cervix (≤25 mm) is reliable in predicting the risk of preterm birth. In addition the efficacy of standard treatments or whether benefit may be derived from prophylactic interventions such as a cervical cerclage is unknown.

**Methods:**

There are three distinct components to the CRAFT project (CRAFT-OBS, CRAFT-RCT and CRAFT-IMG).

CRAFT-OBS: Observational Study; To evaluate subsequent pregnancy risk of preterm birth in women with a prior caesarean section in established labour. This prospective study of cervical length and quantitative fetal fibronectin data will establish a predictive model of preterm birth.

CRAFT-RCT: Randomised controlled trial arm; To assess treatment for short cervix in women at high risk of preterm birth following a fully dilated caesarean section.

CRAFT-IMG: Imaging sub-study; To evaluate the use of MRI and transvaginal ultrasound imaging of micro and macrostructural cervical features which may predispose to preterm birth in women with a previous fully dilated caesarean section, such as scar position and niche.

**Discussion:**

The CRAFT project will quantify the risk of preterm birth or late miscarriage in women with previous in-labour caesarean section, define the best management and shed light on pathological mechanisms so as to improve the care we offer to women and their babies.

**Trial registration:**

CRAFT was prospectively registered on 25th November 2019 with the ISRCTN registry (10.1186/ISRCTN15068651).

**Supplementary Information:**

The online version contains supplementary material available at 10.1186/s12884-020-03375-z.

## Background

Spontaneous preterm birth (sPTB), defined as spontaneous birth less than 37^+ 0^ weeks’ gestation, is a significant health issue and is the most important single determinant of adverse infant outcome with regards to survival and quality of life [1]. Morbidity is inversely correlated to gestational age, and the most significant adverse outcomes are associated with very preterm birth, defined as occurring less than 32^+ 0^ weeks’ gestation. Preventative measures which prolong fetal gestation by 1 week, such as those proposed by this study, could save health services £939 million per year in England and Wales [2].

While factors such as previous sPTB and cervical surgery are known risk factors for sPTB and late miscarriage (LM) [3], recently associations have been reported with full dilatation caesarean sections (FDCS). Emerging evidence has shown an association between late miscarriage (LM) (14–24 weeks’ gestation) and sPTB in women with a FDCS at term (affecting approximately 13.5% of these women), and in women who have had an in-labour caesarean section < 10 cm dilated [4–6]. This is hypothesised to be due to a uterine incision, that is inadvertently too low, in or near to effaced cervical tissue [7]. This occurs because the cervix becomes continuous with the lower segment of the uterus at full dilatation and the anatomy can often be distorted. In addition, tears and angle extensions are common due to the low position of the presenting part [8]. Disruption and scarring of this cervical tissue is thought to result in cervical weakness in future pregnancies. The association between FDCS and LM/sPTB has been recognised in the last 5 years and it presents a pressing clinical problem.

Over 25% of all deliveries in the United Kingdom (UK) are by CS, but up to 20% of in-labour caesarean sections occur at full dilatation, which could affect up to 20,000 women in the UK per annum [9]. These women have a six-fold increased incidence of subsequent preterm birth compared with women who undergo CS in the first stage of labour (adjusted odds ratio 5.8; 95% confidence interval 1.08–30.8) [4]; 13.5% in comparison with 2%. This equates to around 2500 women per year in the UK. In women who lose a pregnancy, more than half will also go on to experience recurrent pregnancy losses in spite of intervention, compared to 14% of women with a history of preterm birth without previous FDCS (relative risk 3.06 95% confidence interval 1.22–7.71) [10]. There is thought to be a continuum of risk, i.e. the later in-labour the CS is carried out, the higher the risk of sPTB in future pregnancies [4].

The insertion of a suture around the cervix under regional anaesthesia is an established management strategy in those women at high risk of sPTB. There is little consensus on the optimal procedure, technique, or timing of insertion. Its mechanism of action has been hypothesised as not only supportive but as reinforcing the immunological barrier which protects the fetus from ascending vaginal infection. The benefit of an ultrasound indicated cerclage following evidence of cervical shortening (≤25 mm) on transvaginal ultrasound scan has been reported to demonstrate a significant reduction in delivery < 35 weeks’ gestation compared with expectant management [11].

A screening programme to assess for cervical shortening in women with a previous FDCS is due to be implemented across England as part of new National Health Service (NHS) commissioning guidance [12] and the revised *Saving Babies Lives Care Bundle* [13]. It is unknown whether transvaginal ultrasound cervical length screening for a short cervix predicts outcome in this group and there is currently limited evidence to inform optimal management of these women. It is also unknown whether first-line interventions for short cervix ≤25 mm (vaginally-placed cerclages inserted in the distal cervix during pregnancy), which is used in other high-risk groups of women, is effective in this cohort. Preliminary data indicates that these interventions may be less efficacious, likely due to the injury having occurred in the proximal cervix, above the cerclage [10].

Currently the ultrasound-assessed cervical length (CL) is the only parameter used in clinical practice to guide management of women at high risk of sPTB and this has not been validated in women with previous FDCS. There is a need to assess the features of the cervix and any scar tissue by both MRI and US to evaluate which characteristics can accurately determine which women are likely to go on to experience LM/sPTB. Preliminary data indicates that scars can be consistently identified on ultrasound and their location defined in relation to the internal-os of the cervix. MRI can provide additional information regarding tissue microstructure. Previous studies have indicated that changes in signal intensity of cervical stromal layers have been found on T2 weighted images [14] and alterations in basic diffusion imaging (sensitive to Brownian motion of water molecules in tissue enabling assessment of microstructure) with relation to the onset of labour reported [15]. This will help evaluate the value of imaging and our ability to predict who will need a cerclage – for example location of scar from FDCS.

The development of sophisticated diffusion-imaging techniques such as Intravoxel Incoherent Motion (IVIM), has enabled assessment of tissue microstructure, diffusivity and perfusion [16]. IVIM separates diffusion perfusion from true diffusion effects, the signal decay is described by a biexponential instead of a mon-exponential model which yields a more accurate description of the underlying tissue properties [17]. This technique has been used to assess perfusion in cervical carcinoma [18] but has not been investigated in relation to sPTB. Professor Daniel Alexander (UCL), a world leading expert in the field of diffusion MR imaging [19], has previously harnessed these techniques in the assessment of the breast [20], brain [21], placenta [22], and prostate [23]. In this project, his team will facilitate optimal data acquisition and analysis of the cervix using IVIM.

The overall aim of the **CRAFT** (**C**erclage **a**fter **f**ull dilatation caesarean sec**t**ion) project.

is to investigate the role of previous in-labour caesarean section in future preterm birth risk and potential management strategies.

## Study design

**Fig. 1:**
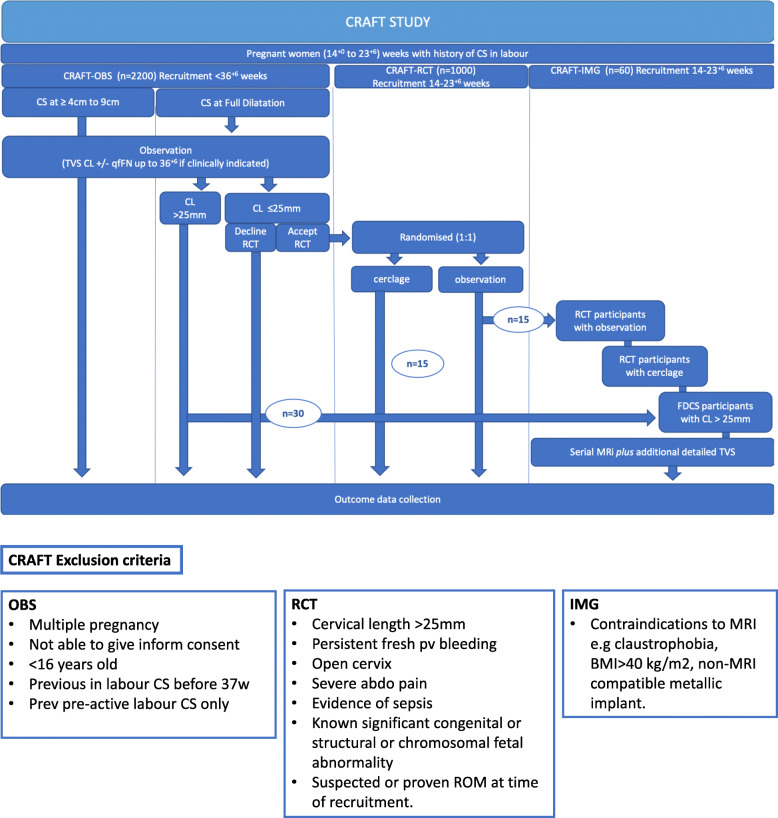
Flow of participants through the CRAFT project. Figure to show the inclusion criteria and flow of participants within the whole CRAFT project (CRAFT-OBS, CRAFT-RCT and CRAFT-IMG)

There will be three components to the CRAFT project (CRAFT-OBS, CRAFT-RCT and CRAFT-IMG). The CRAFT project aims are:

i. To understand the association between the degree of cervical dilatation at CS in-labour with risk of LM/sPTB in subsequent pregnancies (CRAFT-OBS).
ii. To assess the efficacy of cervical cerclage for a short cervix ≤25 mm detected by transvaginal ultrasound in a randomised controlled trial of women with previous FDCS (CRAFT-RCT).
iii. To identify a mechanism for the increased risk of sPTB in women with previous FDCS with Magnetic Resonance Imaging (MRI) and transvaginal ultrasound in order to predict those at most risk and whether cervical cerclage would be of benefit (CRAFT-IMG) (Fig. 1).

### CRAFT-OBS: a study of risk and management in women with a history of CS in-labour

Objectives:

i. Determine the incidence of LM and sPTB (prior to 37 weeks’ gestation) in women with previous CS in-labour stratified by cervical dilatation.
ii. If ultrasound measured cervical length and quantitative fibronectin are carried out clinically, this data will be opportunistically collated and help evaluate the ability of these tests to determine risk of sPTB in women with a history of in-labour caesarean.

This is a multicentre (~ 40 maternity hospitals in the United Kingdom) prospective cohort observational study of 2200 pregnant women with a previous history of term CS in-labour who will be recruited following their booking or scanning visit. Women who have had a previous FDCS and who are found to have a CL ≤25 mm on transvaginal ultrasound will be offered recruitment to the CRAFT-RCT. Women with a previous FDCS and a CL ≤25 mm may be invited to participate in CRAFT-IMG.

### CRAFT-RCT*:* a trial of USS indicated cerclage in women with history of FDCS

Objectives:

i. Determine if an ultrasound-indicated cervical cerclage is effective management in women with a history of FDCS and cervical shortening (≤25 mm) in preventing LM or sPTB < 34 weeks’ gestation. Evaluate the impact of intervention on short-term fetal and neonatal outcomes, assessed as a composite of fetal and perinatal death and major morbidity.
ii. Assess the impact of both management strategies (i.e. cerclage and observation) on health economic outcomes for mother and infant in terms of number of nights in hospital; cost data to hospital discharge/28 days post delivery.

This is a multicentre randomised controlled trial (RCT) in approximately 40 hospitals performing prematurity surveillance (*n* = 1000) to determine whether an ultrasound-indicated cervical cerclage is an effective management option in women with cervical shortening following a FDCS. All women with a history of FDCS will have CL monitoring as per new guidance prior to 24 weeks’ gestation [12]. If their cervical length is or becomes ≤25 mm they will be randomised (1:1) into one of two groups: cervical cerclage plus standard management, or, standard management. The full study design of CRAFT-RCT can be seen in Table 1.

**Table 1:** SPIRIT table for CRAFT-RCT

	STUDY PERIOD					
	Enrolment	Randomisation	Post-allocation			Close-out
TIMEPOINT	***14–23 + 6 weeks’ gestation***	At study recruitment (14–23 + 6 weeks’ gestation)	Within 7 days of randomisation	Antenatal until delivery	Delivery	***28 days post-delivery or discharge from hospital (whichever sooner) of the last recruited participant and infant***
**ENROLMENT:**						
**Eligibility screen**	X					
**Informed consent**	X					
**Allocation**		X				
**INTERVENTIONS:**						
***Insert cervical cerclage***			X			
***Observation***			X			
**ASSESSMENTS:**						
***Appropriate care for as per local unit guidelines***	X	X	X	X	X	
***DATA COLLECTION:***						
***Baseline data (current pregnancy, obstetric and medical history***	X				X	
***Randomisation***		X				
***Pregnancy visit data***		X	X	X		
***Current pregnancy outcomes***					X	
***Data collection complete***						X

CRAFT-RCT will also determine if a shortening cervix predicts outcome in this cohort. If it is demonstrated to be ineffective, further research will be required regarding alternative screening and treatment options.

### CRAFT-IMG: a sub-study evaluating imaging methods for assessing risk and management of women with a history of FDCS

Objectives:

i. To ascertain whether MRI and/or ultrasound findings can predict the risk of sPTB
ii. To identify which women may be most likely to benefit from intervention to prevent sPTB.

Due to MR imaging equipment availability, the CRAFT-IMG subgroup will be recruited from participants attending 2 hospital sites only: University College London and St Thomas’ Hospital, London. All participants will have had a had a prior FDCS and will include women with and without cervical shortening in the current pregnancy based on their CL monitoring. Participants will be allocated to one of three groups: a) women with cervical length of ≤25 mm with no cerclage in place (*n* = 15), b) women with cervical length of ≤25 mm with a cerclage (n = 15), c) women with cervical length of > 25 mm (*n* = 30).

Ultrasound is a commonly used modality in obstetrics to quantify the CL. The CS scar can be identified in the lower uterine segment or cervical tissue using transvaginal ultrasound examination and colour Doppler. The endocervical mucosa and the level of the uterine arteries bilaterally in the para-cervical region is used as a guide to the relative position of the internal cervical os. The CS scar position will be measured in relation to this. The full Caesarean Scar and Niche Measurement Protocol can be found in Additional file 1 [24, 25].

We will compare scar position on MRI and USS in relation to the internal os. We will explore the relationship between scar position, defects in the myometrium and the niche size and shape with adverse pregnancy outcomes such as PTB and LM. The site of previous scar tissue and any other abnormalities (for example the presence of cysts, niches, sonolucent areas, hypoechoic areas and/or anechoic areas [25]) will be recorded where present [26]. Recruited patients will have up to 3 serial transvaginal ultrasounds and MRIs during their pregnancy, as scars are not static and change throughout the course of pregnancy [27]. Scar interpretation therefore must take gestational age into account [27].

### Consent procedure

The study will be verbally explained to potential participants who will be given a written patient information sheet and adequate time for consideration and clarification of any queries. They can take the information home. For CRAFT-OBS and CRAFT-IMG, eligible patients will have until their next appointment to decide on participation and provide informed written consent. For CRAFT-RCT, eligible patients will generally have up to 48 h to decide on participation, depending on the length of their cervix and urgency of treatment as determined by the attending clinician.

Written consent will be confirmed by a clinician or researcher who has undertaken Good Clinical Practice (GCP) training and who is in the local hospital site delegation log (signed by the Investigator at that site). Three copies of the consent form will be taken (1 for participant, 1 for clinical notes (unless electronic records), 1 for site file). At the time of recruitment, a unique study number will be allocated to the patient. Only one copy of patient identifiable data linked to this number will be recorded on a password protected computer in order that recruits can be contacted and delivery outcomes recorded by the local hospital. The research record will contain minimal identifiers such as initials and date of birth. Keeping initials and date of birth on this research database allows the data to be double-checked against source outcome data, therefore enhancing the reliability of the data.

Consent will be sought to obtain NHS numbers for future tracking of outcomes. NHS numbers (mother and baby) will be collected to aid linkage to these future health records as well as follow up pregnancy and neonatal outcomes for recruits who move away from study sites. NHS numbers will be held separately from main study data and only linked with study ID. Access to this information will be limited to specific study staff. They will also be explicitly consented for use of the study data for future research, collection of additional data on any future pregnancies and whether they would be happy to be contacted about participation in further research studies that may be of interest to them.

### Sample size

#### CRAFT-OBS

With estimated prevalence of 15% preterm birth rate prior to 37 weeks’ gestation, and 16.2% of all deliveries being by emergency caesarean section; we will require data on 2200 women to estimate the event rate to 10% relative accuracy (e.g. 13.5 to 16.5%). An average district general hospital has 4000 deliveries per year, of which 648 (16.2%) might be by emergency caesarean. However, considering individual hospital variations of in-labour caesarean rates, workload of clinical research at individual sites, and that not all women approached will wish to take part, the aim is for 40 maternity hospital sites in the United Kingdom to pragmatically achieve our target of 2200 women (approximately 55 recruits per hospital site).

If we acquire data of cervical length and/or quantitative fetal fibronectin in this cohort, we will be able to validate these prediction tools. If we treat a short cervix of ≤25 mm, and a raised qfFN of > = 50 ng/ml as ‘positive tests’, we anticipate a 75% sensitivity and 83% specificity from the literature for both tests [28]. Assuming 10% event rate, 1000 women (100 cases and 900 controls) would allow use to estimate the sensitivities of qfFN and CL to within 10% and the specificities to within 3% of the true value. The positive predictive value would be estimated within 7% and negative predictive value within 1.5% of their true values.

#### CRAFT-RCT

Pilot data indicates that at least 10% of all births and 70% of preterm births associated with FDCS occur before 34 weeks’ gestation. To detect a reduction in this rate from 10 to 5%, 474 women are required in each group to give 80% power. We will aim to recruit 500 in each arm to allow for 5% loss to follow-up.

#### CRAFT-IMG

We consider that the participation of 60 women in this sub-study will provide useful information for planning future research; this is achievable by recruiting at 2 tertiary hospital sites (University College London Hospital and St Thomas’ Hospital, London): 30 women with cervix > 25 mm and 30 women with cervix ≤25 mm (15 women with cerclage, 15 women without).

With cervical length and/or quantitative fetal fibronectin results we will be able to carry out predictive statistics to calculate receiver operating characteristic (ROC) curves for prediction of sPTB for both fibronectin and transvaginal cervical length measurement as a continuous variable, and for delivery < 24, < 30, < 34 and < 37 weeks gestation [29]. From the optimal thresholds, predictive statistics will be calculated. Scar position may be included in the model to aid sPTB prediction.

### Randomisation for CRAFT-RCT

For CRAFT-RCT women will be randomly assigned (1:1) to cerclage or observation at time of recruitment. Computer randomisation will be performed by the co-ordinating research team, and recruiters will be informed the results over the telephone. Due to the nature of the interventions, the study is not blinded to the care providers or patient. Recruiters will not have access to the randomisation sequence. Women will be informed at time of recruitment to which arm they have been randomised. A ‘minimisation’ procedure, using a computer-based algorithm, will be used to avoid chance imbalances in important stratification variables. Stratification variables will be a) previous sPTB, b) previous failed assisted delivery and c) CL of ≤15 mm. Women will not know what treatment they will be allocated prior to recruitment. REDCap, the study specific database management system, will hold a randomisation log accessible by study coordinators and database manager. For more information on the management of data, please see the ‘data management’ section. Contact information will be obtained from the patient. Demographic measures, such as ethnicity and body mass index (BMI), will be entered into the trial database.

Following randomisation in CRAFT-RCT, the attending clinician will arrange for either a cervical cerclage (to be carried out within 7 days) or observation as the randomisation indicates. There is no “emergency code break” procedure as the trial is open label randomised trial. The method and materials used will be according to local protocol, clinician and the individual woman’s preference and documented in the trial database.

Indications for withdrawal of treatment (removal of cerclage) include participant request, elective preterm delivery, fetal membrane rupture, symptomatic placenta praevia and completion to 37 weeks’ gestation. Indication for additional treatment (rescue/emergency cerclage) include exposed membranes or open cervix at 16^+ 0^–27^+ 6^ weeks’ gestation.

### Participant inclusion and exclusion criteria

#### Participant inclusion and exclusion criteria for all CRAFT participants

Inclusion criteria

- Pregnant women up to 36^+ 6^ weeks’ gestation with a history of a previous term (over 37 weeks’ gestation) caesarean section in-labour in any previous pregnancy.
- Current singleton pregnancy.
- Willing and able to give informed consent (with or without interpreter).

Exclusion criteria:

- Under 16 years of age.
- Inability to give informed consent.

#### Participant inclusion and exclusion criteria specific to CRAFT-RCT

Inclusion criteria:

- Pregnant women between 14^+ 0^ and 23^+ 6^ weeks’ gestation with a history of a term (over 37 weeks’ gestation) FDCS.
- Short cervix (≤25 mm) on transvaginal ultrasound scan.

Exclusion criteria:

- Women with persistent fresh vaginal bleeding evident on speculum examination.
- Women with visible fetal membranes evident on speculum examination or open cervix on ultrasound scan.
- Women with severe abdominal pain/evidence of sepsis (as judged by attending clinician).
- Known significant congenital or structural or chromosomal fetal abnormality.
- Suspected or proven rupture of the fetal membranes at the time of recruitment.
- Women who have any cerclage in situ.

#### Participant inclusion and exclusion criteria specific to CRAFT-IMG

Inclusion criteria:

- Pregnant women between 14^+ 0^ and 23^+ 6^ weeks’ gestation with a history of term FDCS.

Exclusion criteria:

- Contraindications to MRI, e.g. claustrophobia, BMI > 40 kg/m2 (due to technical limitations of scanner) or non-MRI compatible metallic implants.

### Data analysis

Data will be analysed using Stata software Version 15 or later (StataCorp, College Station, Texas). Statistical analysis of results will be undertaken by the research team and the project statistican, Mr. Paul Seed.

#### CRAFT-OBS

This study will calculate prediction of preterm birth < 37 weeks’ gestation in women with a previous CS in-labour by observing clinical pregnancy outcomes. Where data is available the predictive value based on cervical length and quantitative fetal fibronectin will also be assessed. Standard predictive statistics will be used for prediction of delivery < 34 and < 37 weeks’ gestation (sensitivity, specificity, positive predictive value, negative predictive values, and ROC areas for continuous measures).

Standard predictive statistics will be used for prediction of delivery < 34 weeks gestation (cervix ≤25 mm will be calculated along with area under the curve). We will also validate our current predictive tools of cervical length and fetal fibronectin in this population in this population (including the QUiPP mobile phone app [29]).

#### CRAFT-RCT

The main analyses will be by intention to treat. The primary outcome is sPTB under 34 weeks.

Secondary analyses will include the following endpoints:

i. Adverse perinatal outcome, defined as composite outcome of death (antepartum or intrapartum stillbirths or neonatal deaths prior to discharge) or one/more of intraventricular haemorrhage, periventricular leukomalacia, hypoxic ischaemic encephalopathy, necrotizing enterocolitis, bronchopulmonary dysplasia and sepsis. The components of the composite will also be presented.
ii. Gestation at delivery.
iii. Requirement for rescue cerclage (exposed fetal membranes).
iv. Time between intervention and delivery.
v. Health costs at 28 days post-delivery.

Results will be presented as both risk ratios and risk differences, leading to number needed to treat (NNT) if appropriate, according to CONSORT guidelines. A subgroup analysis will be carried out according to other risk factors for preterm birth other than FDCS.

#### CRAFT-IMG

This sub-study will provide data on relationships between structural uterine and cervical defects in pregnant women who have had a previous FDCS and a short cervix ≤25 mm, compared with those who have a cervix > 25 mm. Comparison will be made between the two groups. Relative efficiency of imaging the scar and its associated defects by MRI (and relationships with outcomes), will be compared to those identified by ultrasound in order to ascertain whether there is any correlation in findings between the two modalities [14, 15]. We will also assess changes in the cervix and uterus over time. Images will be assessed for overt structural abnormalities in collaboration with radiologists using a structured proforma. Patients and clinicians will be informed of any clinically significant findings (for example, placenta previa or placenta accreta).

### Missing data, unused data and false data

#### Missing data

The main analysis will adjust for bias due to missing data under the missing at random assumption using multiple regression.

We will follow a four-point framework for handling observations that are incomplete, allowing the appropriate method to be chosen and subsequently implemented [30].

1. Attempt to follow up all randomized participants, even if they withdraw from allocated treatment.
2. Perform a main analysis of all observed data that is valid under a plausible assumption about the missing data. Specifically, we will assume data is missing at random. Under this assumption, imbalances between treatment groups due to dropout can be corrected by appropriate multiple regression models.
3. Perform a sensitivity analyses to explore the effect of departures from the assumption made in the main analysis. The missing not at random analysis will use the method of White et al. (2011) as implemented in the Stata command rctmiss.
4. Account for all randomized participants, at least in the sensitivity analyses.

This framework details the significance of using credible assumptions when handling missing data. For the main analysis we will assume that missing data is missing at random and the effect of the intervention is equivalent in those with and without the observations. We will then verify whether there is an imbalance or if the percentage of missing data is comparable within each treatment allocation.

#### Unused data

We will follow the intention to treat principle for analysis. All consenting women randomised with sufficient data collected will be included in the primary and main secondary endpoints.

#### False data

Reasonable precautions will be undertaken to minimise the number of data errors. Those inputting data on the study database will have appropriate training as outlined under Data Collection. All data entered will be examined and queries raised if necessary. Firstly by the coordinating research team once local sites have entered it on the database, and secondly by the trial statistican at time of analysis.

### Data collection

Following consent, data on the participant’s demographic characteristics, risk factors, medical and obstetric history will be documented and entered onto the study specific REDcap database (https://externalredcap.isd.kcl.ac.uk/). This will include current and previous monitoring procedures and/or pregnancy interventions (e.g. cervical length measurements and cerclage). Gestational age will be calculated using ultrasound estimated date of delivery predicted at the 12–15 week ultrasound scan. Pregnancy and neonatal outcomes will be collected from NHS patient records postnatally. Those with access to inputting data on the study database will have to complete test participants before gaining full access to ensure they are trained appropriately.

Participants with a history of term FDCS will be referred to a preterm surveillance clinic where they will be offered cervical length assessment (with or without other tests, such as fetal fibronectin) from 14^+ 0^ to 23^+ 6^ weeks gestation as per current standard care pathway. Cervical length will be measured with transvaginal ultrasound in accordance with local protocols, measurements will be taken in triplicate and the shortest measurement used. If the cervical length is, or becomes ≤25 mm during this period the participant will be offered the opportunity to take part in CRAFT-RCT. Further explanation and a separate information sheet will be provided and additional written consent will be obtained. If the participant is unwilling to participate in this randomised controlled trial she will continue to be monitored and cared for as per local protocol/clinicians’ experience and continue in the observation arm of the CRAFT study. Women who provide written informed consent will be randomised to either cervical cerclage or observation. Both arms will receive follow-up as per local guidelines.

Sixty participants will be invited to have additional imaging tests for the sub-study, CRAFT-IMG. They will be given additional information and asked to sign another consent form. CRAFT-IMG involves serial transvaginal ultrasound measurements of cervical length (which they may already be offered as part of standard care, utilising the protocol methodology described in Additional file 1), and up to 3 MRI scans of the lower uterus and cervical structure (which are not part of standard care).

Serial MRI scans will be performed on a 3-Tesla MRI scanner at St Thomas’ Hospital. Conventional imaging will include high resolution T1 and T2 weighted images, MR relaxometry and MRI diffusion. The first advanced protocol transvaginal ultrasound and MRI will occur at any time from 14 weeks’ gestation (which is the lower gestation limit for CRAFT-IMG recruitment). Where delivery has not occurred, further imaging will be offered (up to a maximum of 3 MRI and 3 transvaginal ultrasound scans).

Outcome data: Participant data will be collected up to discharge following delivery. Neonatal outcomes will be collected up to discharge or 28 days (whichever is sooner). Prompts on the database will alert the research midwife/assistant when each trial participant reaches her delivery date. Birth registers and in-patient records will be used to track hospital admissions and pregnancy outcomes. Outcome data (medical and healthcare utilisation) will be collected by review of NHS maternity and medical records. If information is unavailable, e.g. if the delivery occurred elsewhere, the patient, patient’s GP or other hospital will be contacted. A full list of specific outcomes and the measures utilised can be seen in Additional file 2.

### Data management

All records will be managed to ensure they are GDPR compliant. A bespoke internet-based data management system will be designed, built and maintained by REDCap. Secure access to the database is granted to authorised users through individual log-in and password. Following recruitment, a unique study number will be allocated to the participant. Contact details, linked only by study ID, will be kept separately on secure local hospital site computers.

Paper copies of consent forms will be stored numerically (by study ID) and kept in a locked filing cabinet at local hospital sites. NHS numbers (mother and baby) will be collected to follow up pregnancy and neonatal outcomes for recruits who move away from study sites. Access to this information will be limited to specific study staff.

The co-ordinating research team and the study statistican, Mr. Paul Seed, will have access to the full final trial dataset.

### Auditing

Meetings will be held on a regular basis by the co-ordinating research team to monitor and audit the conduct of the research and review aspects of the CRAFT study’s progress. The individual hospital site Investigator(s) will permit trial-related monitoring and audits by providing the co-ordinating research team with access to source data and other documents (e.g. patients’ case sheets, MRI reports etc). This process by the co-ordinating research team will be independent from Investigators or the Sponsor(s).

### Safety

All Adverse Events (AEs) and Serious adverse Events (SAEs) must be recorded from the time a participant is randomized to treatment until 30 days after stopping the intervention and until pregnancy outcome (28 days after delivery). Open-ended and non-leading verbal questioning of the participant should be used to enquire about AE/SAE occurrence at every visit during the study. If there is any doubt as to whether a clinical observation is an AE/SAE, the event should be recorded. Hospitalisations for treatment planned prior to randomisation and hospitalisation for elective treatment of a pre-existing condition will not be considered as an SAE. Complications occurring during such hospitalisation will be AE/SAEs.

Expected serious adverse events are those events which are expected in the patient population or as a result of the routine care/treatment of a patient. The interventions they will be receiving are those which would be offered routinely in clinical practice. Cervical cerclage insertion is an established surgical procedure, which is associated with minimal risks. These include infection, miscarriage, bleeding, difficulty with suture removal and preterm prelabour rupture of membranes. SAEs and serious adverse reactions (SARs) which are unrelated to these clinical procedures will be reported as SAEs.

Events that are primary or secondary outcome measures are not considered to be SAEs and will be reported in the normal way, on the appropriate REDCap outcome form.

Maternal:

- Premature labour
- Premature rupture of membranes
- Chorioamnionitis

Infant:

- Perinatal death (unless unexpected at this gestation)
- Low birth weight
- Requirement for supplemental oxygen or ventilation support
- Complications of prematurity (e.g. IVH, NEC, encephalopathy, seizures, hypoglycaemia) unless unexpected in this population
- Admission of the baby to the neonatal unit

In addition, the following common pregnancy complication events will not be considered SAEs: hospitalisation for pre-eclampsia or pregnancy induced hypertension, hospitalisation for symptoms of preterm labour (e.g. rupture of membranes, vaginal bleeding); hospitalisation for maternal discomfort; hospitalisation for rest; hospitalisation for observation or monitoring for which the woman is admitted for a period of less than 12 h; delivery complications such as caesarean section or postpartum haemorrhage.

All SAEs (excepting those specified in this protocol as not requiring reporting) will be reported immediately (and certainly no later than 24 h) by the Investigator at the study hospital site to the sponsor Research and Development Office and the CRAFT Chief Investigator for review in accordance with the current Pharmacovigilance Policy. The Chief Investigator will report to the relevant ethics committee.

### Trial steering committee

The Trial Steering Committee (TSC) will monitor the progress of the study and conduct and advise on its scientific credibility. The TSC will consider (and act, as appropriate), upon the recommendations of the Data Monitoring Committee (DMC). The TSC ultimately carries the responsibility for deciding whether the trial needs to be stopped on the grounds of safety or efficacy. The TSC will consist of an independent chair and at least two other independent members (not involved in study recruitment and not employed by any organisation directly involved in study conduct). The first meeting will take place 6 months after trial start date; frequency will be decided at the first meeting (at least annually).

### Data monitoring committee

The DMC will consist of a chair and at least two other independent members. The committee will periodically review study progress and outcomes as well as reports of serious adverse events (SAEs). The DMC will, if appropriate, make recommendations regarding the continuance of the study or modification of the study protocol, and provide advice to the TSC. The frequency (if any) of interim analyses will be determined by the DMC, supported by the study statistican Mr. Paul Seed. The DMC will meet 3 months following study commencement; frequency of meeting will be decided at the first meeting (at least annually).

### Study stopping rules

CRAFT may be prematurely discontinued by the TSC, Sponsor, Chief Investigator or Regulatory Authority on the basis of new safety information or for other reasons given by regulatory or ethics committees concerned. If the trial is prematurely discontinued, active participants will be informed and no further participant data will be collected. The Competent Authority and Research Ethics Committee will be informed within 15 days of the early termination of the trial.

### Protocol and other study document amendments

Any changes in research activity, except those necessary to remove an apparent, immediate hazard to the participant, must be reviewed and approved by the Chief Investigator and the Co-Sponsors notified. Substantial amendments to the protocol and other study documents must be submitted by the co-ordinating research team in writing to the appropriate REC, Regulatory Authority and local R&D for approval prior to participants being enrolled into an amended protocol. Communication of amendments will be via email. Any amendments will only be implemented following Ethics Committee and Trust approval by the R&D Department in each participating hospital site.

### Expected study duration and COVID-19

Recruitment was due to be conducted over a 24 month period with the total study length of 30 months. However like many research studies, recruitment was paused during the COVID-19 pandemic in 2020 to enable clinical research staff to focus on COVID-19 research and/or be redeployed to clinical services. Contemporaneous information on amendments to extend study recruitment during this rapidly changing situation can be found on our ISRCTN webpage: http://www.isrctn.com/ISRCTN15068651

The end of the study will be defined as 28 days post-delivery or discharge from hospital (whichever sooner) of the last recruited participant and infant. The end of the study will be reported to the Research Ethics Committee (REC) and Regulatory Authority within 90 days, or 15 days if the study is terminated prematurely.

### Dissemination policy

Results of the study will be presented at national and international conferences and reported in peer reviewed journals. No patient identifiable information will be published. A lay summary of the results will be presented back to the King’s College London/ Guy’s and St Thomas’ Preterm Birth Patient and Public Involvement Group, alongside being available for participants via the study website (https://www.kcl.ac.uk/research/craft).

### Insurance and indemnity

This study is co-sponsored by King’s College London and Guy’s and St Thomas’ NHS Foundation Trust. The sponsors will at all times maintain adequate insurance in relation to the study independently. King’s College London, through its own professional indemnity (Clinical Trials) and no-fault compensation and the Trust having a duty of care to patients via NHS indemnity cover, in respect of any claims arising as a result of clinical negligence by its employees, brought by or on behalf of a study patient.

## Discussion

Despite emerging evidence on the association between FDCS and the risk of sPTB/LM, many uncertainties remain, including the degree of risk following an in-labour CS, before full dilatation. Our decision support tool, the QUiPP app [29, 31], utilises predictive models based on transvaginal ultrasound and quantitative fetal fibronectin, but these are not validated for use in this specific cohort of women. It is unknown whether the commonly used clinical threshold of cervical length ≤ 25 mm is a reliable predictor of sPTB in women with a history of in-labour CS. These women may benefit from prophylactic treatment, such as placement of cervical cerclage before, rather than after, the cervix has started to shorten. Certain defects are visible on MRI and ultrasound but whether this is of use clinically remains unclear, and could be of benefit in understanding the mechanism of LM and sPTB following CS. The CRAFT project will investigate the role of previous in-labour caesarean section as a risk factor for future sPTB, and will inform the development of management strategies to optimise the care we can offer to this recently identified group of high risk women.

## Supplementary Information

**Additional file 1.** Caesarean Scar and Niche Measurement Protocol for the CRAFT study.

**Additional file 2.**

## Data Availability

Data sharing is not applicable to this protocol manuscript as no datasets have been generated or analysed yet.

## Abbreviations

BMI: Body Mass Index
CL: Cervical length
CS: Caesarean section
FDCS: Fully dilated caesarean section
IVIM: Intravoxel Incoherent Motion
LM: Late miscarriage
MRI: Magnetic Resonance Imaging
NHS: National Health Service
qfFN: Quantitative fetal fibronectin
RCT: Randomised control trial
REC: Research Ethics Committee
sPTB: Spontaneous preterm Birth
TAC: Transabdominal cerclage
UK: United Kingdom
