# Supplementary material for: CRAFT (Cerclage after full dilatation caesarean section): protocol of a mixed methods study investigating the role of previous in-labour caesarean section in preterm birth risk
Source: BMC Pregnancy Childbirth. 2020 Nov 16;20:698. doi: 10.1186/s12884-020-03375-z (PMC7667480; doi:10.1186/s12884-020-03375-z)
Supplement: Supplementary file 1 — Additional file 1. Caesarean Scar and Niche Measurement Protocol for the CRAFT study. [file 12884_2020_3375_MOESM1_ESM.docx]

**Appendix 1: Caesarean Scar and Niche Measurement Protocol for the CRAFT study**

**Definitions**

Caesarean Scar - hypoechoic (or hyperechoic) discontinuity in the myometrium at the anterior wall of the lower uterine segment or cervix.

Caesarean Niche - an indentation at the site of the caesarean scar.

**Measurements**

- To measure the caesarean scar and niche, there should be good visualisation of the lower uterine segment and cervix. After identifying the CS scar, magnify the image so the scar occupies at least 50% of the image to ensure consistent and accurate measurements.
- Use the uterine arteries and the endocervical mucosa as a guide to the relative position of the internal cervical os.
  - - To identify the uterine artery:
      - Apply color flow mapping
      - Move the transducer laterally to view the para-cervical region
      - Gently move the transducer from side to side to identify the uterine artery and the level at which it straightens out (aliasing vessels coursing along the side of the cervix and uterus)
- In the sagittal plane record distance between the base of the caesarean scar and the internal cervical os.
- Measurements of niche in sagittal plane (Figure 1A) include: length, depth, residual myometrial thickness (RMT) and adjacent myometrial thickness (AMT) (1,2)
- Measurement of niche in the transverse plane (Figure 1B) include: width
- If present, niche branches should be reported and additional measurements undertaken.
- Further measurements include distance between niche and vesicovaginal (VV) fold and volume of cervix.

Figure 1A: Figure 1B:


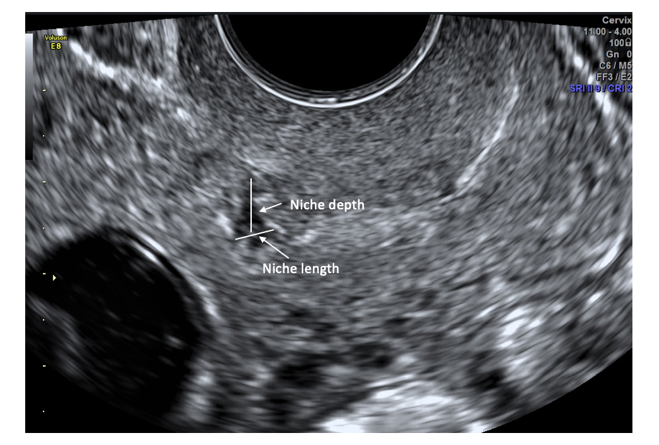

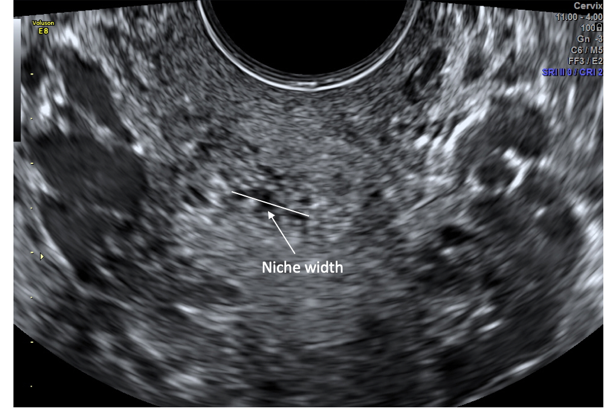


References:

1. Naji O, Abdallah Y, Bij De Vaate AJ, Smith A, Pexsters A, Stalder C, et al. Standardized approach for imaging and measuring Cesarean section scars using ultrasonography. Ultrasound Obstet Gynecol [Internet]. 2012 Mar 1;39(3):252–9. Available from: https://doi.org/10.1002/uog.10077

2. Jordans IPM, de Leeuw RA, Stegwee SI, Amso NN, Barri-Soldevila PN, van den Bosch T, et al. Sonographic examination of uterine niche in non-pregnant women: a modified Delphi procedure. Ultrasound Obstet Gynecol [Internet]. 2019 Jan 1;53(1):107–15. Available from: https://doi.org/10.1002/uog.19049
