# Supplementary material for: CRAFT (Cerclage after full dilatation caesarean section): protocol of a mixed methods study investigating the role of previous in-labour caesarean section in preterm birth risk
Source: BMC Pregnancy Childbirth. 2020 Nov 16;20:698. doi: 10.1186/s12884-020-03375-z (PMC7667480; doi:10.1186/s12884-020-03375-z)
Supplement: Supplementary file 2 — Additional file 2. [file 12884_2020_3375_MOESM2_ESM.pdf]

Enrolment

Study ID ______________________

Estimated Delivery Date ______________________

Registration

Age at Enrolment ______________________

Height (cm) ______________________

Weight (kg) ______________________

Ethnicity:
- ◯ European
- ◯ Indian
- ◯ Pakistani
- ◯ Bangladeshi
- ◯ Black Caribbean
- ◯ Black African
- ◯ Middle Eastern
- ◯ Far East Asian
- ◯ Unclassified (other)

Ethnicity other: ______________________

Comments

____

Eligibility For Observation

Eligibility CRAFT-Observation
Inclusion criteria

Date of recruitment ________________________________

Gestation at recruitment (weeks) ________________________________

Gestation at recruitment (days) ________________________________

Pregnant < = 36+6 weeks gestation
◯ Yes
◯ No

Singleton pregnancy
◯ Yes
◯ No

Willing and able to give informed consent (with or without interpreter)
◯ Yes
◯ No

Any previous caesarean section in labour (include if dilated but value unknown) AT TERM
◯ Yes
◯ No

Caesarean details

Is cervical dilatation known
◯ Yes
◯ No

Cervical dilation at time of emergency caesarean (cm) ________________________________

Unit where Caesarean in labour occurred:

◯ Airedale NHS Foundation Trust
◯ Aneurin Bevan University Health Board
◯ Ashford and St. Peter's Hospitals NHS Foundation Trust
◯ Barking, Havering and Redbridge Hospitals NHS Trust
◯ Barts Health NHS Trust
◯ Basildon and Thurrock University Hospitals NHS Foundation Trust Website.
◯ Betsi Cadwaladr University Health Board
◯ Birmingham Women's and Children's NHS Foundation Trust
◯ Blackpool Teaching Hospitals NHS Foundation Trust
◯ Bolton NHS Foundation Trust
◯ Bradford Teaching Hospital NHS Foundation Trust
◯ Brighton and Sussex University
◯ Buckinghamshire Healthcare NHS Trust
◯ Burton Hospitals NHS Foundation Trust
◯ Calderdale and Huddersfield NHS Foundation Trust
◯ Cambridge University Hospitals NHS Foundation Trust
◯ Cardiff and Vale University Health Board
◯ Central Manchester University Hospital NHS Trust
◯ Chelsea and Westminster Hospital NHS Foundation Trust (Chelsea and Westminster Hospital)
◯ Chelsea and Westminster Hospital NHS Foundation Trust (West Middlesex University Hospital)
◯ Countess of Chester NHS Foundation Trust
◯ County Durham and Darlington Foundation Trust
◯ Dartford and Gravesham NHS Trust
◯ Durham and Darlington NHS Trust
◯ East Kent Hospitals University NHS Foundation Trust (Queen Elizabeth The Queen Mother Hospital)
◯ East Kent Hospitals University NHS Foundation Trust (William Harvey Hospital)
◯ East Lancashire Hospitals NHS Trust
◯ Epsom and St Helier University Hospitals NHS Trust
◯ Exeter and Devon NHS Trust
◯ Frimley Health NHS Foundation Trust
◯ Guy's and St Thomas' NHS Foundation Trust
◯ Heart of England NHS Foundation Trust
◯ Hillingdon Hospitals NHS Foundation Trust
◯ Imperial College Healthcare NHS Trust
◯ Kettering General Hospital NHS Trust
◯ Kingston Hospital NHS Foundation Trust
◯ Lancashire Teaching Hospitals NHS Foundation Trust
◯ Leeds Teaching Hospitals NHS Trust
◯ Lewisham and Greenwich NHS Trust (Queen Elizabeth Hospital)
◯ Lewisham and Greenwich NHS Trust (University Hospital Lewisham)
◯ Liverpool Women's NHS Foundation Trust
◯ London North West University Healthcare NHS Trust (Ealing Hospital)
◯ London North West University Healthcare NHS Trust (Northwick Park Hospital)
◯ Luton and Dunstable University Hospital NHS Foundation Trust
◯ Maidestone and Tunbridge Wells NHS Trust
◯ Mid Cheshire Hospitals NHS Foundation Trust
◯ Mid Essex Hospital Services NHS Trust
◯ Mid Yorkshire Hospitals NHS Trust
◯ Milton Keynes University Hospital NHS Foundation Trust
◯ Newcastle upon Tyne Hospitals NHS Foundation Trust
◯ NHS Fife
◯ NHS Grampian
◯ NHS Greater Glasgow and Clyde
◯ NHS Lothian
◯ Norfolk and Norwich University Hospitals NHS Foundation Trust
◯ North Bristol NHS Trust

◯ North Middlesex University Hospital
◯ Northern Devon Healthcare NHS Trust
◯ NorthWest Anglia NHS Foundation Trust
◯ Nottingham University Hospitals NHS Foundation Trust
◯ Oxford University Hospitals NHS Foundation Trust
◯ Pennine Acute Hospitals NHS Trust (North Manchester General Hospital)
◯ Pennine Acute Hospitals NHS Trust (Royal Oldham Hospital)
◯ Poole Hospital NHS Foundation Trust
◯ Portsmouth Hospitals NHS Trust
◯ Princess Alexandra Hospital NHS Trust
◯ Royal Berkshire NHS Foundation Trust
◯ Royal Cornwall Hospitals NHS Trust
◯ Royal Devon and Exeter NHS Foundation Trust
◯ Royal Free London NHS Foundation Trust
◯ Royal United Hospitals Bath NHS Foundation Trust
◯ Royal Wolverhampton NHS Trust
◯ Sheffield Teaching Hospitals NHS Foundation Trust
◯ South Tyneside and Sunderland NHS Foundation Trust
◯ Swansea Bay University Health Board
◯ The Dudley Group NHS Foundation Trust
◯ The Royal Wolverhampton NHS Foundation Trust
◯ The Shrewsbury and Telford Hospital NHS Trust
◯ United Lincolnshire Hospitals NHS Trust
◯ University College London Hospitals NHS Foundation Trust
◯ University Hospital Southampton NHS Foundation Trust
◯ University Hospitals Bristol NHS Foundation Trust
◯ University Hospitals Coventry and Warwickshire NHS Trust
◯ University Hospitals of Derby and Burton NHS Foundation Trust
◯ University Hospitals of Leicester NHS Trust
◯ University Hospitals of North Midlands NHS Trust
◯ University Hospitals Plymouth NHS Trust
◯ University Hospital Southampton NHS Foundation Trust
◯ Warrington and Halton Hospitals NHS Foundation Trust
◯ Western Sussex University Hospitals NHS Trust
◯ Whittington Health NHS Trust
◯ Wirral University Teaching Hospital NHS Foundation Trust
◯ Worcestershire Acute Hospitals NHS Trust
◯ Wye Valley NHS Trust
◯ York Teaching Hospital NHS Foundation Trust
◯ Other (please specify)

Other location for caesarean (please specify) __________________________________

Verification of dilatation documentation
◯ Yes
◯ No

This participant may be eligible for randomisation, please check eligibility criteria

Exclusion criteria

< 16 years old

◯ Yes
◯ No

Summary

Eligible

◯ Yes
◯ No

Reason for non-eligibility

____

Comments

____

Pregnancy History

Parity

No of pregnancies ending < 13+6 weeks ________________

No of pregnancies ending 14-23+6 weeks ________________

No of pregnancies ending 24+0 - 36+6 weeks ________________

No of pregnancies ending >37+0 weeks ________________

Other details

____

Comments

____

Obstetric History (one form per pregnancy ongoing > 24 weeks)

Is this the pregnancy which made you eligible? (Caesarean delivery in labour and as close to 10cm dilated or at 10cm exactly if more than one)

◯ Yes
◯ No

Year (of delivery) ________________________________

Gestational age at delivery (weeks) ________________________________

Gestational age at delivery (days) ________________________________

Onset of Labour

◯ Spontaneous
◯ Induced
◯ Caesarean Section
◯ Other (give details below)

(NOTE: Option " Caesarean Section" must not be selected if you have answered 'yes' to 'Is this the pregnancy which made you eligible?')

Other details (onset of labour):

____

Mode of Delivery

◯ Spontaneous
◯ Assisted
◯ Caesarean Section
◯ Surgical management of miscarriage
◯ Other (give details below)

Other details (mode of delivery):

____

Suspected infection details if applicable

Catheterised?

◯ Unknown
◯ Yes
◯ No

Evidence of urinary retention in labour

◯ Unknown
◯ Yes
◯ No

Pyrexia in labour?

◯ Unknown
◯ Yes
◯ No
◯ N/A

Pyrexia post partum?

◯ Unknown
◯ Yes
◯ No

Need for antibiotics due to suspicion of infection?

◯ Unknown
◯ Yes
◯ No

Histological evidence of chorioamnionitis?
- ◯ Unknown
- ◯ Yes
- ◯ No

Microbiological evidence of chorioamnionitis?
- ◯ Unknown
- ◯ Yes
- ◯ No

Labour and delivery details (provide what is available)

Was a caesarean section performed?
- ◯ Yes
- ◯ No

Was labour established (>= 4cm)?
- ◯ Yes
- ◯ No
- ◯ N/A

Did the membranes rupture before caesarean?
- ◯ Unknown
- ◯ Yes
- ◯ No

SROM or ARM?
- ◯ SROM
- ◯ ARM

Date of rupture of membranes ______________________

Time of rupture of membranes ______________________

Cervical dilatation at time of emergency caesarean (cm) ______________________

Date and time of established labour ______________________

Date and time of established 2nd stage ______________________

Time commenced pushing ______________________

Date commenced pushing ______________________

Date and time of decision for trial in theatre or CS ______________________

Length (minutes) of first stage ______________________

Length (minutes) of second stage ______________________

Date and time of delivery ______________________

Caesarean section details (provide what is available)

Seniority of surgeon
- ◯ SHO
- ◯ ST3-5
- ◯ ST6-7
- ◯ Post CCT fellow
- ◯ Consultant
- ◯ Unknown

Failed trial?
- ◯ Ventouse
- ◯ Forceps (outlet)
- ◯ Forceps (rotational)
- ◯ No attempts

Type of incision
- ◯ Lower segment transverse
- ◯ J shaped
- ◯ Inverted T
- ◯ Vertical
- ◯ Unknown

Any inadvertent uterine extensions?
- ◯ Yes
- ◯ No

Where was this extension located?
- ☐ Superiorly
- ☐ Inferliorly
- ☐ Left angle
- ☐ Right angle
- ☐ Midline inferiorly
- ☐ Extending into the broad ligament
- ☐ Extending posteriorly

Size of most significant extension (mm) ________________________________

Evidence of impacted head?
- ◯ Yes
- ◯ No

Number of haemostatic sutures ________________________________

Double or single layer uterine closure
- ◯ Single
- ◯ Double

Suture used to close uterine incision
- ◯ Vicryl
- ◯ Dexon
- ◯ Catgut
- ◯ Other (please specify)

Other suture details ________________________________

Delivery details

Number of fetuses
- ◯ singleton
- ◯ twins
- ◯ triplets

Estimated blood loss
- ◯ Unknown
- ◯ < = 500ml
- ◯ >500ml and < 1000ml
- ◯ >= 1000ml

Number of units blood transfused:
- ◯ 0
- ◯ 1
- ◯ 2
- ◯ 3
- ◯ 4
- ◯ 5
- ◯ 6
- ◯ >6

Comments

Medical History Risk Factors

Medical History (pre-existing Medical conditions at time of enrolment)

Pre-existing hypertension:	◯ Unknown<br>◯ Yes<br>◯ No
Asthma:	◯ Unknown<br>◯ Yes<br>◯ No
Type 1 Diabetes:	◯ Unknown<br>◯ Yes<br>◯ No
Type 2 Diabetes:	◯ Unknown<br>◯ Yes<br>◯ No
Autoimmune disease:	◯ Unknown<br>◯ Yes<br>◯ No
Chronic renal disease:	◯ Unknown<br>◯ Yes<br>◯ No
Chronic viral infection:	◯ Unknown<br>◯ Yes<br>◯ No
Lupus antibodies confirmed positive:	◯ Unknown<br>◯ Yes<br>◯ No
Antiphospholipid syndrome confirmed positive:	◯ Unknown<br>◯ Yes<br>◯ No
Other:	◯ Yes<br>◯ No
If other please specify:	__________________________________

Current medications at the time of enrolment

Antihypertensives	◯ Unknown<br>◯ Yes<br>◯ No
If yes, please specify antihypertensives:	__________________________________
Steroids	◯ Unknown<br>◯ Yes<br>◯ No

If yes, please specify steroids: __________________________________

Immunosuppressive agents
- ◯ Unknown
- ◯ Yes
- ◯ No

If yes, please specify immunosuppressive agents:
- ◯ Steroids
- ◯ Azathioprine
- ◯ Tacrolimus
- ◯ MMF (mycophenolate mofetil)
- ◯ Sulfasalazine
- ◯ Other

Please specify other immunosuppressive __________________________________

Antibiotics
- ◯ Unknown
- ◯ Yes
- ◯ No

If yes, please specify antibiotics: __________________________________

Other medications
- ◯ Unknown
- ◯ Yes
- ◯ No

Other medications (please specify) __________________________________

Preterm birth risk factors

Previous spontaneous preterm birth
- ◯ Unknown
- ◯ Yes
- ◯ No

Previous midtrimester loss
- ◯ Unknown
- ◯ Yes
- ◯ No

Previous PPROM (Preterm Pre-Labour Rupture of Membranes)
- ◯ Unknown
- ◯ Yes
- ◯ No

Cervical History

Previous Cervical Surgery?
- ◯ No
- ◯ Once
- ◯ Twice

Year of 1st cervical surgery __________________________________

Type of 1st cervical surgery
- ◯ Cone
- ◯ LLETZ
- ◯ Trachelectomy
- ◯ Cervical biopsy
- ◯ Unknown

Anaesthetic (1st cervical surgery)
- ◯ General Anaesthetic
- ◯ Local Anaesthetic

Depth (1st cervical surgery)
- ◯ < 10mm
- ◯ >= 10mm
- ◯ Unknown

Year of 2nd cervical surgery ______________________________

Type of 2nd cervical surgery
- ◯ Cone
- ◯ LLETZ
- ◯ Trachelectomy
- ◯ Cervical biopsy
- ◯ Unknown

Anaesthetic (2nd cervical surgery)
- ◯ General Anaesthetic
- ◯ Local Anaesthetic

Depth (2nd cervical surgery)
- ◯ < 10mm
- ◯ >=10mm
- ◯ Unknown

History of 2 or more proven recurrent UTIs in pregnancy:
- ◯ Unknown
- ◯ Yes
- ◯ No

Past or present history of GBS
- ◯ Unknown
- ◯ Yes
- ◯ No

Past or present history of domestic violence:
- ◯ Yes
- ◯ No
- ◯ Unknown

Past or present history of recreational drug use:
- ◯ Unknown
- ◯ Yes
- ◯ No

Smoking:
- ◯ Never
- ◯ Ex
- ◯ Current

If yes to smoking, how many per day? ______________________________

Uterine abnormality:
- ◯ Unknown
- ◯ None
- ◯ Double cervix
- ◯ Submucosal fibroids
- ◯ Bicornuate uterus
- ◯ Intra-uterine septum
- ◯ Other

Other details: ______________________________

Comments

____

Current Pregnancy Visit (one per preterm surveillance visit)

Current ongoing pregnancy: Asymptomatic screening

TVUS for cervical length? ◯ Yes ◯ No

TVUS date ____________________________

Gestational age (weeks) ____________________________

Gestational age (days) ____________________________

What is 1st cervical length (mm) ____________________________

What is 2nd cervical length (mm) ____________________________

What is 3rd cervical length (mm) ____________________________

Clinically indicated fFN? ◯ Yes ◯ No

Result qfFN (ng/mL) ____________________________

Comments ____________________________________

Current Pregnancy Outcomes (OBS and RCT)

Current ongoing pregnancy: Labour details

Date of hospital admission ________________

Onset of labour
- ◯ Spontaneous
- ◯ Induced
- ◯ Pre-labour Caesarean

If spontaneous onset of labour was it augmented?
- ◯ Yes
- ◯ No

If NOT spontaneous onset of labour reason for induction or pre-labour C-section
- ◯ Pre-eclampsia
- ◯ Obstetric cholestasis
- ◯ Pre-existing/gestational diabetes
- ◯ Antepartum haemorrhage
- ◯ Maternal infection
- ◯ Other maternal medical
- ◯ Suspected fetal growth restriction
- ◯ Other suspected fetal compromise
- ◯ Post dates
- ◯ Malpresentation
- ◯ Pre-labour ruptured membraness
- ◯ Previous caesarian section
- ◯ Maternal request
- ◯ Other

Other reason for induction please specify ________________

Antibiotics in labour
- ◯ Yes
- ◯ No

Antibiotic name(s) Max of three
- ☐ Co-amoxiclav
- ☐ Amoxicillin
- ☐ cefalexin
- ☐ Benzylpenicillin
- ☐ Gentamicin
- ☐ Nitrofurantoin
- ☐ Trimethoprim
- ☐ Vancomycin
- ☐ Clindamycin
- ☐ Other

Other antibiotic please specify: ________________

Pregnancy Preterm Interventions

Transvaginal cerclage inserted during pregnancy?
- ◯ Yes
- ◯ No

Date of transvaginal cerclage insertion ________________

Date of transvaginal cerclage removal ________________

Was it a difficult cerclage removal
- ◯ Yes
- ◯ No

Arabin pessary inserted during pregnancy?
◯ Yes
◯ No

Date of arabin pessary insertion ______________________

Date of arabin pessary removal ______________________

Commenced on progesterone in pregnancy?
◯ Yes
◯ No

Date progesterone commenced ______________________

Date progesterone ended ______________________

Type of progesterone
◯ IM
◯ Oral
◯ PV
◯ PR

Total daily dose of progesterone (mg) ______________________

Ultrasound indication or history indication for progesterone
◯ Ultrasound indication
◯ History indication

Was there evidence in labour of:

Maternal pyrexia (>37.5C):
◯ Yes
◯ No
◯ Unknown

Raised CRP >10 mg/L:
◯ Yes
◯ No
◯ Unknown

Please specify highest CRP value recorded: ______________________

Raised white blood cell count (WBC):
◯ Yes
◯ No
◯ Unknown

Please specify highest WBC value recorded (10 _9/L): ______________________

Positive MSU
◯ Yes
◯ No
◯ Unknown

Positive HVS
◯ Yes
◯ No
◯ Unknown

Positive blood culture
◯ Yes
◯ No
◯ Unknown

Chorioamnionitis confirmed on histopathology
◯ Yes
◯ No
◯ Unknown

Date of maternal discharge: ______________________

Total inpatient postnatal nights: ______________________

Comments

____

Maternal Postnatal Details

Total number of unscheduled suspected pre-term birth antenatal day unit attendances ______________________________

Total number of antenatal inpatient nights ______________________________

Date of ruptured membranes: ______________________________

Time of ruptured membranes: ______________________________

Blood loss ml
- ◯ < 500ml
- ◯ >=500ml
- ◯ >=1000ml
- ◯ Unknown

(ml)

Mothers age at time of delivery (years) ______________________________

Neonatal Outcomes

Date of delivery: ______________________________

Time of delivery: ______________________________

Mode of Delivery
- ◯ Spontaneous
- ◯ Assisted
- ◯ Elective Caesarean Section
- ◯ Emergency Caesarean (latent phase)
- ◯ Emergency Caesarean (labour)
- ◯ SMM
- ◯ Other (give details below)

Other details (mode of delivery):

____

Gestation at delivery (weeks) ______________________________
(weeks)

Gestation at delivery (days) ______________________________
(days)

Pregnancy outcome
- ◯ Live birth
- ◯ Stillbirth
- ◯ Late miscarriage
- ◯ Neonatal death

Sex of baby
- ◯ Male
- ◯ Female

Birthweight (g) ______________________________

APGAR score 1 minute: ______________________________

APGAR score 5 minutes: ____________________

Major congenital abnormality
◯ Yes
◯ No

Details of Congenital abnormality ____________________

Admission to SCBU or NICU
◯ Yes
◯ No

If suspected preterm birth

At any point in this pregnancy did she receive tocolysis
◯ Yes
◯ No

Date received tocolysis ____________________

Tocolytic
◯ Atosiban
◯ Nifedipine
◯ Indomethacin
◯ Salbutamol
◯ other

Gestational age given (weeks) ____________________ ((weeks))

Gestational age given (days) ____________________ ((days))

Number of days tocolysis ____________________

Magnesium sulphate for neuroprotection
◯ Yes
◯ No

Gestation of magnesium sulphate (weeks) ____________________ (weeks)

Gestation of magnesium sulphate (days) ____________________ (days)

Duration of magnesium sulphate (hours) ____________________ (hours)

Duration of magnesium sulphate (min, round to nearest 30 min)
◯ 0
◯ 30
(min)

Received steroids for fetal lung maturation?
◯ Yes
◯ No

Date received first steroid ____________________

Steroid
◯ Betamethasone
◯ Dexamethasone
◯ other/unsure

Gestational age given (weeks) ____________________ ((weeks))

Gestational age given (days) ______________________ ((days))

Full course
◯ Yes
◯ No

Baby Complications

Respiratory distress syndrome
◯ Yes
◯ No

Pulmonary hypertension of the newborn
◯ Yes
◯ No

Meconium aspiration syndrome
◯ Yes
◯ No

Retinopathy of prematurity
◯ Yes
◯ No

Intraventricular haemorrhage
◯ Yes
◯ No

Oxygen at 28 days
◯ Yes
◯ No

Positive kleihauer
◯ Yes
◯ No

Thrombocytopenia
◯ Yes
◯ No

Necrotising enterocolitis
◯ Yes
◯ No

Pneumothorax
◯ Yes
◯ No

Hypoxic ischaemic encephalopathy
◯ Yes
◯ No

USS brain abnormality
◯ Yes
◯ No

Still in neonatal unit at 28 days?
◯ Yes
◯ No

Positive culture of infection in the first 48hours
◯ Yes
◯ No

Number of inpatient nights ______________________

Neonatal death date: ______________________

End report

Pregnancy outcome status

- ◯ Yes known >24 weeks
- ◯ Pregnancy ended < 24 weeks
- ◯ Outcome unknown

Comments

____

Primary pregnancy outcomes

Spontaneous onset of labour resulting in delivery 24 - 36+6 weeks	◯ Yes<br>◯ No
Spontaneous onset of labour resulting in delivery 24 - 33+6 weeks	◯ Yes<br>◯ No
Spontaneous onset of labour resulting in delivery 14 - 23+6 weeks	◯ Yes<br>◯ No
Spontaneous onset of labour resulting in delivery < 13+6 weeks	◯ Yes<br>◯ No
Premature prelabour rupture of membranes	◯ Yes<br>◯ No

Other pregnancy complications

Pre-eclampsia	◯ Yes<br>◯ No
SGA	◯ Yes<br>◯ No
Obstetric cholestasis	◯ Yes<br>◯ No
Gestational diabetes	◯ Yes<br>◯ No
Antepartum haemorrhage	◯ Yes<br>◯ No
Maternal death	◯ Yes<br>◯ No
Pregnancy complications other	◯ Yes<br>◯ No

Comments

____

If outcome unknown

Is there missing outcome data?
- ◯ Yes
- ◯ No

Number of attempts to contact patient __________________________________

Contacted by phone
- ◯ Yes
- ◯ No
- ◯ N/A

Contacted by email
- ◯ Yes
- ◯ No
- ◯ N/A

Contacted GP
- ◯ Yes
- ◯ No
- ◯ N/A

Contact through NHS tracing service attempted
- ◯ N/A
- ◯ Once
- ◯ Twice
- ◯ More than twice

Maternal outcome comment

____

Eligibility For RCT

CRAFT-RCT Eligibility

Inclusion criteria

Pregnant between 14+0 and 23+6 weeks gestation	◯ Yes<br>◯ No
Previous term FDCS	◯ Yes<br>◯ No
Singleton pregnancy	◯ Yes<br>◯ No
Short cervix (< =25mm) on TVUS	◯ Yes<br>◯ No
Willing and able to give informed consent (with or without interpreter)	◯ Yes<br>◯ No

Exclusion criteria

< 16 years old	◯ Yes<br>◯ No
Fresh PV bleeding on speculum examination	◯ Yes<br>◯ No
Visible fetal membranes on speculum examination or open cervix on ultrasound	◯ Yes<br>◯ No
Severe abdominal pain	◯ Yes<br>◯ No
Suspected or proven ruptured fetal membranes	◯ Yes<br>◯ No
Suspected sepsis	◯ Yes<br>◯ No
Known significant congenital, structural or chromosomal fetal abnormality	◯ Yes<br>◯ No
Cerclage in situ	◯ Yes<br>◯ No

Eligible for RCT?

Eligible for RCT?	◯ Yes<br>◯ No
Offered RCT?	◯ Yes<br>◯ No
Reason for not offering RCT	◯ Carrying more than one baby<br>◯ Doesn't meet eligibility criteria<br>◯ Meets exclusion criteria<br>◯ Unable to provide informed consent<br>◯ Does not want to take part<br>◯ Unacceptable to clinician<br>◯ Other please specify in comments box
Accepted RCT?	◯ Yes<br>◯ No
If not accepted, specify reason	◯ Unable to provide informed consent<br>◯ Does not want to take part<br>◯ Unacceptable to clinician<br>◯ Contra-indication to MRI if IMG eligible<br>◯ Other please specify in comments box
Comments	__________________________________

Randomisation CRAFT-RCT

Date of Consent ______________________________

Date of randomisation ______________________________

Cervical length
- ◯ CL< =15mm
- ◯ CL>15mm

Did the patient have a failed assisted delivery
- ◯ Yes
- ◯ No

Did the patient have a previous spontaneous preterm birth prior to 37 weeks gestation.
- ◯ Yes
- ◯ No

Only add centre here if delivered elsewhere ______________________________

Gestational age at time of randomisation (weeks) ______________________________

Gestational age at time of randomisation (days) ______________________________

Are you requesting randomisation
- ◯ Yes
- ◯ No

Please contact the central team by phone to randomise on 07557815039

Randomised to
- ◯ Cerclage
- ◯ No cerclage

Comments

____

Current Pregnancy Visits CRAFT-RCT (one per visit)

Date of visit ______________________

Gestational age (weeks) ______________________

Gestational age (days) ______________________

CRAFT-RCT consent signed
- ◯ Yes
- ◯ No

Reason for visit
- ◯ Follow-up
- ◯ Symptoms
- ◯ Reassurance
- ◯ Due to inpatient stay
- ◯ Other

Other please specify ______________________

Prescribed progesterone in this pregnancy
- ◯ Yes
- ◯ No

Prescribed arabin pessary this pregnancy?
- ◯ Yes
- ◯ No

New medications prescribed at this visit?
- ◯ Yes
- ◯ No

If yes specify formulation
- ☐ Aspirin
- ☐ Levothyroxine
- ☐ Folic acid
- ☐ Vitamin D
- ☐ Paroxetine
- ☐ Sertraline
- ☐ Citalopram
- ☐ Dexamethasone
- ☐ Salbutamol inhaler
- ☐ Labetalol
- ☐ Nifedipine
- ☐ Methyldopa
- ☐ Paracetamol
- ☐ Codeine/dihydrocodeine
- ☐ Progesterone
- ☐ Promethazine
- ☐ Prochlorperazine
- ☐ Metoclopramide
- ☐ Cyclizine
- ☐ Ondansetron
- ☐ Ranitidine
- ☐ Omeprazole
- ☐ Gaviscon
- ☐ Piriton (chlorphenamine)
- ☐ Cetirizine
- ☐ Lactulose
- ☐ Senna
- ☐ Isphagula/fybogel
- ☐ Other

Participation in other research since consented
- ◯ Yes
- ◯ No

Concurrent studies
- ☐ Insight
- ☐ C-Stitch
- ☐ C-Stitch 2
- ☐ Other

Other concurrent study (please specify) ______________________________

Currently on antibiotics
- ◯ Yes
- ◯ No

If currently on antibiotics, please specify
- ☐ Amoxicillin
- ☐ Cephalexin
- ☐ Nitrofurantoin
- ☐ Co-amoxiclav
- ☐ Erythromycin
- ☐ Other please specify

Please specify other antibiotic: ______________________________

Vaginal pessary in previous 24 hours
- ◯ Yes
- ◯ No

If yes, specify pessary
- ◯ Progesterone
- ◯ Clotrimazole
- ◯ Metronidazole
- ◯ non-medicated (for prolapse)
- ◯ Other

Vaginal douching in previous 24 hours
- ◯ Yes
- ◯ No

Sexual intercourse in previous 24 hours
- ◯ Yes
- ◯ No

Sexual intercourse in previous 48 hours
- ◯ Yes
- ◯ No

Surveillance of cervical length

Cervical length performed?
- ◯ Yes
- ◯ No
- ◯ Unsure

What is 1st cervical length (mm) ______________________________

What is 2nd cervical length (mm) ______________________________

What is 3rd cervical length (mm) ______________________________

Membranes bulging
- ◯ Yes
- ◯ No
- ◯ Unsure

Funnelling
- ◯ Yes
- ◯ No

Height of funnel (mm) ______________________________

Width of funnel (mm) ______________________________

Sludge
- ◯ Yes
- ◯ No

Placenta clear of os
- ◯ Yes
- ◯ No
- ◯ Unsure

Pressure effect when suprapubic pressure applied?
- ◯ Yes
- ◯ No

fFN performed?
- ◯ Yes
- ◯ No

fFN results: ______________________________
((ng/mL))

Cerclage details

Was a cerclage inserted
- ◯ Yes
- ◯ No

What type of cerclage was inserted?
- ◯ Macdonald
- ◯ Shirodkar
- ◯ Rescue

Material used
- ◯ monofilament
- ◯ braided

What type of anaesthetic was used for cerclage insertion
- ◯ GA
- ◯ spinal
- ◯ epidural

When was the cerclage inserted? ______________________________

When was the cerclage removed? ______________________________

Gestation when cerclage removed (weeks) ______________________________

Gestation when cerclage removed (days) ______________________________

Was the insertion supervised by a Consultant?
- ◯ Yes
- ◯ No

What was the seniority of the surgeon inserting the cerclage?
- ◯ Consultant
- ◯ Post CCT Fellow
- ◯ Specialist Fellow
- ◯ Sub-speciality Trainee
- ◯ Registrar
- ◯ Senior Registrar
- ◯ Staff Grade
- ◯ SHO

Was it a difficult removal?
- ◯ Yes
- ◯ No

What type of anaesthetic was used for cerclage removal?
- ◯ LA
- ◯ GA
- ◯ Regional
- ◯ None

Side effects of management with cerclage, arabin pessary and/or progesterone

What type of management is in place?
- ☐ None
- ☐ Cerclage
- ☐ Arabin
- ☐ Progesterone

(Select all that apply)

Lower abdominal pain
- ◯ Yes
- ◯ No

Vaginal discomfort/pain
- ◯ Yes
- ◯ No

Vaginal bleeding
- ◯ Yes
- ◯ No

Difficulty voiding urine
- ◯ Yes
- ◯ No

Difficulty with defaecation
- ◯ Yes
- ◯ No

Increased vaginal discharge
- ◯ Yes
- ◯ No

Other side effects
- ◯ Yes
- ◯ No

If other side effects, please specify ________________________________

Did the woman request removal of treatment/alternative
- ◯ Yes
- ◯ No

If woman requested removal of treatment/alternative, record reason ________________________________

Was alternative treatment prescribed this visit
- ◯ Yes
- ◯ No

If yes, which
- ◯ Cerclage
- ◯ Progesterone
- ◯ Arabin pessary

Inpatient Care:

Total number of unscheduled antenatal day unit attendances so far for suspected preterm birth/concerns ________________________________

Inpatient currently?
- ◯ Yes
- ◯ No

If yes, how many days in total? ________________________________

Inpatient reason
- ◯ Suspected preterm birth
- ◯ PPROM
- ◯ Hypertension
- ◯ Suspected pre-eclampsia
- ◯ Reduced fetal movements
- ◯ antepartum haemorrhage
- ◯ Unstable lie
- ◯ Induction of labour
- ◯ Suspected infection
- ◯ Other maternal medical problem
- ◯ Concern about fetal growth/wellbeing
- ◯ Social reasons

Total number of antenatal inpatient nights: ______________________

Follow-up plan: ______________________

Follow-up plan weeks ______________________

Transferred to routine antenatal care this visit?
- ◯ Yes
- ◯ No

Between 34+0 and 37+0 weeks
- ◯ Yes
- ◯ No

Date for removal of cerclage booked if applicable? ______________________

Comments ______________________

RCT Adverse Events

Adverse Event (please give diagnosis if known) __________________________________

Body system code
- ◯ Cardiovascular
- ◯ Respiratory
- ◯ Hepatic
- ◯ Gastro-intestinal
- ◯ Genito-urinary
- ◯ Endocrine
- ◯ Haematological
- ◯ Musculo-skeletal
- ◯ Neoplasia
- ◯ Neurological
- ◯ Psychological
- ◯ Immunological
- ◯ Dermatological
- ◯ Allergy
- ◯ ear,nose,throat
- ◯ Other

If other, please specify ______________________________

Start date ______________________________

Stop date ______________________________

Expected AE
- ◯ Yes
- ◯ No

CTCAE severity grade (version 5.0)
- ◯ Grade 1
- ◯ Grade 2
- ◯ Grade 3
- ◯ Grade 4
- ◯ Grade 5

Relationship to study treatment
- ◯ None
- ◯ Unlikely
- ◯ Possible
- ◯ Likely
- ◯ Definite

Indication ______________________________

Comments __________________________________

Eligibility For Imaging

CRAFT-IMG Eligibility

Inclusion criteria

Pregnant between 14+0 and 23+6 weeks gestation at recruitment	◯ Yes<br>◯ No
Previous term FDCS	◯ Yes<br>◯ No
Singleton pregnancy	◯ Yes<br>◯ No
Willing and able to give informed consent (with or without interpreter)	◯ Yes<br>◯ No

Exclusion criteria

< 16 years old	◯ Yes<br>◯ No
Unable to give informed consent	◯ Yes<br>◯ No
Only previous caesarean section carried out before labour	◯ Yes<br>◯ No
Claustrophobic	◯ Yes<br>◯ No
BMI>40	◯ Yes<br>◯ No
MRI contraindication (e.g. metallic implant)	◯ Yes<br>◯ No

Eligible for imaging?

Eligible for imaging?	◯ Yes<br>◯ No
Offered Imaging?	◯ Yes<br>◯ No
Reason for not offering imaging	◯ Carrying more than one baby<br>◯ Doesn't meet eligibility criteria<br>◯ Meets exclusion criteria<br>◯ Unable to provide informed consent<br>◯ Does not want to take part<br>◯ Unacceptable to clinician<br>◯ Other please specify in comments box

Accepted Imaging?	◯ Yes<br>◯ No
Reason for not accepting imaging	◯ Unable to provide informed consent<br>◯ Does not want to take part<br>◯ Unacceptable to clinician<br>◯ Contra-indication to MRI if IMG eligible<br>◯ Other please specify in comments box
Comments	__________________________________

MRI

Day of Imaging

Date of MRI scan ________________________

Raw data number ________________________

Gestational age at day of imaging (weeks) ________________________

Gestational age at day of imaging (days) ________________________

Transvaginal cervical cerclage in situ
◯ Yes
◯ No

If yes, date transvaginal cervical cerclage inserted ________________________

Temperature before MRI ________________________
(Range 35.5-37.5, 1 d.p)

Temperature before MRI ________________________

Temperature after MRI ________________________
(Range 35.5-37.5, 1 d.p)

Temperature after MRI ________________________

MRI clinical report

____

MRI parameters

Validation number ________________________

CBD number ________________________

T2 cor uterus ________________________

T2 sag uterus ________________________

T2ME ________________________

T1DIR ________________________

T2ME-ASL ________________________

Comments

____

TVUS

Date of TVUS ________________

Gestational age at day of TVUS (weeks) ________________

Gestational age at day of TVUS (days) ________________

Transvaginal cervical cerclage in situ
- ◯ Yes
- ◯ No

If yes, date transvaginal cervical cerclage inserted ________________

Total number of cervical cysts ________________

Is CS scar visible?
- ◯ Yes
- ◯ No

Distance from base of CS scar to internal cervical os (mm) ________________
(Input number accurate to 1 decimal place)

Shortest distance from base of CS scar to internal cervical os (mm) ________________
(Input number accurate to 1 decimal place)

Presence of niche/defect?
- ◯ Yes
- ◯ No

Dimensions of defect length (mm) ________________

Dimensions of defect width (mm) ________________

Dimensions of defect depth (mm) ________________

Residual myometrial thickness (RMT) mm ________________

Residual myometrial thickness (AMT) mm ________________

Is funnelling present?
- ◯ Yes
- ◯ No

Branches present?
- ◯ Yes
- ◯ No

Length to internal os (mm) ________________

Width of funnel (mm) ________________

Sludge
- ◯ Yes
- ◯ No

Has had steroids
- ◯ Yes
- ◯ No

Gestation at time of steroids (weeks) ________________

Full course
- ◯ Yes
- ◯ No

Has had antibiotics in last 2 weeks
- ◯ Yes
- ◯ No

Gestation at time of antibiotics (weeks)

◯ 12
◯ 13
◯ 14
◯ 14
◯ 15
◯ 16
◯ 17
◯ 18
◯ 19
◯ 20
◯ 21
◯ 22
◯ 23
◯ 24
◯ 25
◯ 26
◯ 27
◯ 28
◯ 29
◯ 30
◯ 31
◯ 32
◯ 33
◯ 34
◯ 35
◯ 36
◯ N/A

Reason ________________________________

TVUS clinical report: ____

Comments ____

Randomisation log

Requestor name ______________________________

Meets eligibility criteria for CRAFT-RCT
◯ Yes
◯ No

Cervical Length
◯ < =15mm
◯ >15mm

Previous spontaneous preterm birth < 37 weeks?
◯ Yes
◯ No

Previous failed assisted delivery?
◯ Yes
◯ No

Date/Time of Minim randomisation ______________________________

Coordinator performing randomisation
◯ Naomi Carlisle
◯ Giorgia Dalla Valle
◯ Debbie Finucane
◯ Agnieszka Glazewska-hallin
◯ Holly Lovell
◯ Vicky Robinson
◯ Andrew Shennan
◯ Lisa Story
◯ Natalie Suff

Randomised to
◯ Cerclage
◯ No cerclage

Randomisation email sent?
◯ Yes
◯ No

Comments

____
